# FIR-LSTM: An Explainable Deep Learning Framework for Predicting Iatrogenic Withdrawal Syndrome in Pediatric Intensive Care Units

**DOI:** 10.21203/rs.3.rs-6787167/v1

**Published:** 2025-06-25

**Authors:** Haoqiu Song, Anita K. Patel, Murray M. Pollack, Layne T. Watson, Liqing Zhang

**Affiliations:** 1 Department of Computer Science, Virginia Polytechnic Institute and State University, Blacksburg, VA, USA; 2 Fralin Biomedical Research Institute, Virginia Tech FBRI Cancer Research Center, Washington, D.C., USA; 3 Division of Critical Care Medicine, Children’s National Health System, Washington, D.C., USA; 4 Department of Pediatrics, George Washington University School of Medicine and Health Sciences, Washington, D.C., USA

**Keywords:** Iatrogenic Withdrawal Syndrome, Explainable AI, Electronic Health Records, Critical Care, Multivariate Time Series Prediction

## Abstract

Iatrogenic withdrawal syndrome (IWS) is a significant yet underrecognized public health concern for pediatric patients in critical care units, most frequently the result of abrupt cessation or rapid tapering of sedative or opioid medications. Early prediction of IWS is important for timely intervention and improved patient outcomes. In this study, we developed an explainable deep learning model utilizing a unidirectional multilayer long short-term memory (LSTM) network to predict the risk of IWS in pediatric ICU patients. Through longitudinal electronic health records (EHRs), our model analyzes the preceding 24 hours of patient data to predict the likelihood of IWS occurring in the next four hours, providing a real-time risk score. To enhance interpretability and identify key risk factors, we applied layer-wise relevance propagation (LRP) to the LSTM model. The feature importance rankings derived from LRP were validated through multiple experiments. Experimental results show that the model was perfectly calibrated and achieved robust predictive performance, suggesting that the LRP enhanced LSTM model holds significant potential for improving pediatric patient care by facilitating early detection and proactive management of IWS in critical care settings. Implementing this model into a system of alerts for clinicians could lead to significant advances in safer sedative and analgesic use, addressing an under addressed public health issue that impacts not only the United States but also the global community.

## Introduction

Iatrogenic withdrawal syndrome (IWS) is a significant yet underrecognized complication in pediatric critical care medicine. In the medical environment, it often occurs when sedatives and/or opioids are being weaned or stopped, particularly after their prolonged use. These medications are commonly administered in intensive care units (ICUs) to manage pain and anxiety^1,2^. Despite advancements in critical care, IWS affects up to 65% of children who receive sedatives and/or opioids for longer than five days, contributing to delayed recovery, increased healthcare costs, significant distress for both patients and their families, prolonged hospitalization, and protracted treatment with long-acting opioid and sedative medications such as methadone and lorazepam^3–9^. Early identification and proactive management of IWS are important for mitigating these negative impacts. However, current clinical practices often rely on observational assessments and subjective scoring systems; no studies have focused on the dynamic risk of withdrawal throughout a patient’s ICU stay despite the known association between IWS and prolonged exposure to sedative and opioid medications^10^. Pediatric IWS is often identified using validated screening tools including the Withdrawal Assessment Tool-1 (WAT-1) and the Sophia Observation Withdrawal Symptoms Scale (SOS) ^4,11–13^. The WAT-1 score (detail is provided in Appendix A), the most widely used tool in North America, is an 11 item, 12-point scale with a score greater than or equal to three indicating the IWS^6,11^. However, since this system relies heavily on subjective judgements and manual screening tools, there is a pressing need for reliable, automated, and data driven tools that can assist clinicians in identifying patients at high risk of IWS early in their treatment course, which can benefit not only the United States but also the whole world.

Electronic health records (EHRs) offer a rich source of longitudinal patient data that can be harnessed for predictive analytics^14^. Advances in machine learning, particularly deep learning techniques, have shown promise in modeling complex temporal patterns within EHR data for various clinical predictions^15^. Long short-term memory (LSTM) networks, a type of recurrent neural network, are well suited for handling time series data and capturing dependencies over extended periods^16^. However, the “black box” nature of deep learning models poses challenges for clinical adoption, as clinicians require transparency and interpretability to trust and act upon model predictions^17^.

Explainable artificial intelligence (XAI) methods aim to address this challenge by providing insights into the decision-making processes of complex models^18^. Layer-wise relevance propagation (LRP) is an XAI technique that can attribute the output of a neural network to its input features, offering a way to understand which variables most influence the model’s predictions^19^. Incorporating interpretability into predictive models is essential for aligning machine learning advancements with clinical needs and fostering trust among healthcare providers^20^.

In this study, we present FIR-LSTM, an explainable deep learning framework designed to predict IWS risk in pediatric ICU patients using longitudinal EHRs and medication data. Previous applications of LRP in conjunction with LSTM models (e.g., Bi-LSTM or attention based variants)^21–26^ have largely focused on natural language processing tasks. By contrast, our model targets a clinical time series setting, integrating continuous and discrete variables, such as vital signs, medication usage, and other temporal information, to produce real-time risk scores for IWS. A key innovation of FIR-LSTM lies in its unidirectional multilayer LSTM architecture, which leverages the preceding 24 hours of patient data to forecast the likelihood of IWS in the subsequent 4-hour window. This design ensures that future time epochs do not influence prior time epochs, preserving critical causal structures in the data. The 24-hour horizon provides ample medication history, while the 4-hour prediction interval aligns with standard clinical assessments of pediatric vital signs such as heart rate and respiratory rate. Crucially, by applying LRP to each prediction, FIR-LSTM offers transparent attributions for the input variables contributing to IWS risk, thus bridging the gap between complex deep learning methods and clinician interpretability.

To validate both performance and interpretability, we conducted rigorous, permutation-based tests and applied our model to a new independent cohort. These evaluations confirm that the identified risk factors and their temporal significance are both statistically valid and clinically meaningful. To our knowledge, this work represents the first systematic approach to estimating IWS risk in a pediatric population using a combination of advanced time series modeling, comprehensive EHR data integration, and state-of-the-art interpretability techniques. Ultimately, FIR-LSTM has the potential to assist clinicians in proactively identifying and managing at risk children, thereby contributing to improved patient care outcomes.

## Methodology

### Data Preprocessing

**Figure 1.**
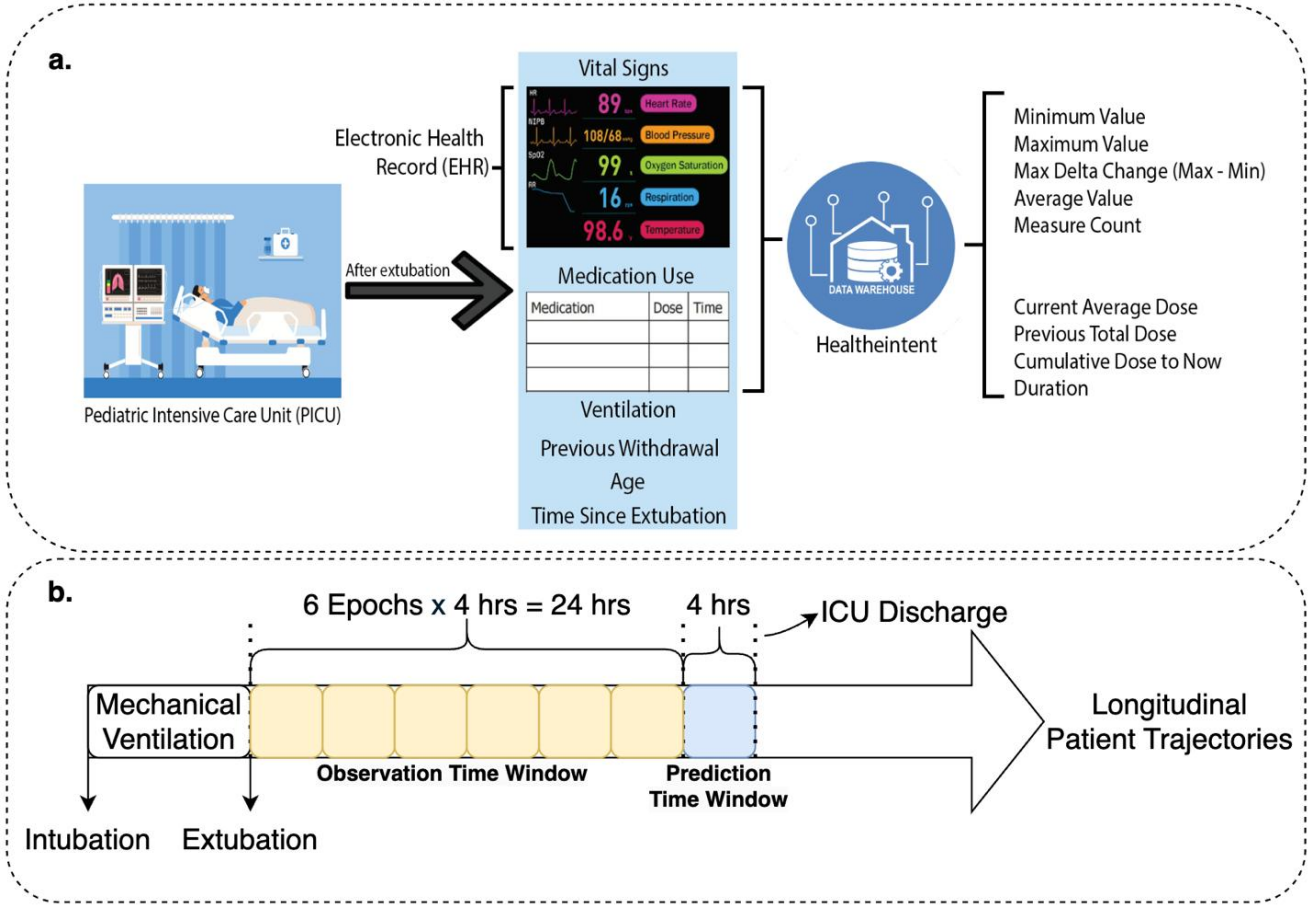
Overview of data preprocessing process. (a). Schematic of data extraction from the pediatric intensive care unit (PICU) EHR, including vital signs and medication usage. Summarized features (e.g., min, max, average values, dose metrics) are computed for each 4-hour epoch. (b) Construction of the 24-hour observation window (six epochs of four hours each) and the subsequent 4-hour prediction window for modeling IWS risk.

Figure 1 outlines the data preprocessing steps. The initial stage of data preprocessing involved constructing a structured, time resolved feature set from the EHR data. We focused on capturing both physiological trends and medication administration patterns that could be predictive of IWS. The overall goal was to transform dynamic patient information into a standardized sequence of feature vectors amenable to machine learning.

We began by identifying the patient population of interest: pediatric patients managed in the pediatric and pediatric cardiac intensive care units (collectively referred to as PICU). We considered the period following cessation of mechanical ventilation as the starting point for our analyses. Transitions off mechanical support represent a critical time window for the potential emergence of withdrawal symptoms as medications are weaned and/or discontinued. For each patient, we collated all relevant EHR data, including vital signs and medication records, over a 24-hour “observation” time window. The 24-hour time window was segmented into six discrete 4-hour epochs to provide a granular, longitudinal representation of clinical trajectories. To capture the dynamic progression of a patient’s condition over time, we applied a sliding window approach whereby the starting point of each 24-hour window was shifted forward by one epoch for subsequent data analyses, thereby enabling the model to learn from multiple, partially overlapping segments of an individual patient’s clinical course.

Within each 4-hour time epoch, we extracted and aggregated multiple types of features from the raw EHR signals. Detailed descriptions of the features are provided in Appendix B. For vital signs, including temperature, heart rate, systolic blood pressure, diastolic blood pressure, respiratory rate, and mean arterial pressure, we computed summary statistics that characterized both central tendencies and dynamic fluctuations within each 4-hour time epoch. Specifically, we recorded the minimum and maximum observed values, the max delta change (i.e., the difference between the maximum and minimum), the average value across the epoch, and the number of measurements collected. This approach captured not only the prevailing physiological state but also the variability within that epoch.

Medication administration data were processed to derive temporal administration patterns. For each individual sedative and opioid analgesic medications, we computed the current average dose per kilogram over the 4-hour window, the cumulative dose per kilogram administered up to that point in time, and the previous total dose per kilogram administered in the previous 4-hour time epoch. Additionally, the cumulative duration of exposure to these medications was captured. By quantifying medication use in these standardized forms, we transformed potentially irregular dosing schedules into normalized features reflecting both magnitude and history of drug exposure. These features were merged along with other relevant variables, such as patient age, documented prior withdrawal episodes, and duration of mechanical ventilation to form the input vectors. Each input vector represented a 4-hour snapshot of the patient’s clinical state containing 66 features in total, resulting in a sequence of six consecutive 4-hour time epochs spanning the previous 24-hour observation window. Following this feature extraction process, we aligned each patient’s data to predict risk of IWS in the subsequent 4-hour time epoch. Patient time periods without the requisite preceding 24-hour observation window were not eligible to make a future risk of IWS prediction.

Finally, we performed standard data cleaning and imputation to ensure a robust and uniform dataset for subsequent analyses. Among the vital sign features, the proportion of missing values ranged from 0.16% for respiratory rate to 17.19% for mean arterial pressure, with intermediate missing rates for temperature (0.22%), heart rate (0.74%), systolic blood pressure (12.65%), and diastolic blood pressure (12.66%). Missing observations were imputed using a patient-centric approach: when available, the most recent valid measurement within the previous four hours was carried forward; otherwise, the patient’s mean value across all available measurements was used. For the current average dose of each medication, any interval lacking documentation was set to zero, indicating no dosage administered during that period. After applying these imputation strategies, no missing values remained in the dataset. In addition, continuous variables were rescaled to the [0,1] range via Min-Max scaling to prevent any single feature from dominating the model training. The resulting time series comprised 24-hour observation windows for each patient, aligned with a subsequent 4-hour prediction interval, serving as the input for our downstream modeling pipeline.

### Model Architecture

**Figure 2.**
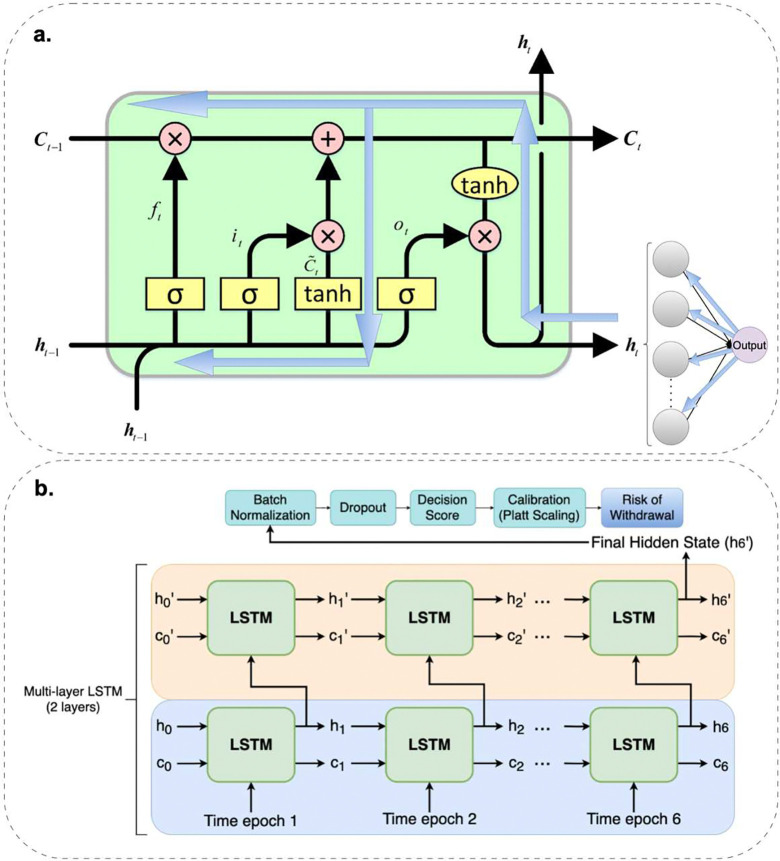
Architecture of FIR-LSTM. (a). Schematic of the unidirectional LSTM cell and its gating mechanism. The superimposed blue arrows represent the backpropagation of relevance scores via LRP, illustrating how feature importance is determined through tracing back to earlier time steps. (Adapted from https://stackoverflow.com/questions/50488427/what-is-the-architecture-behind-the-keras-lstm-cell.) (b). Overview of a two-layer LSTM model architecture to generate a risk score for IWS.

In this study, we propose a predictive modeling framework, FIR-LSTM, a unidirectional, multilayer LSTM architecture trained to anticipate the onset of IWS based on a 24-hour clinical history. This approach leverages the LSTM’s capacity to model long range temporal dependencies inherent in the time series nature of EHR data and integrates an LRP algorithm to ensure interpretability of the model’s predictions. Figure 2 shows the architecture of FIR-LSTM, including the single LSTM cell with LRP and the overview of the multilayer structure.

To be more specific, the LSTM model was designed to operate on sequential patient level data, where each sequence comprised six discrete and consecutive time epochs, each representing four hours of patient observations and corresponding feature sets. The LSTM layer maintains a hidden state and a cell state at each time step. The hidden and cell states are updated according to

(1) $$i_{t}=\sigma \left(W_{ih}^{\left(i\right)}x_{t}+W_{hh}^{\left(i\right)}h_{t-1}+b^{\left(i\right)}\right),$$

(2) $$f_{t}=\sigma \left(W_{ih}^{\left(f\right)}x_{t}+W_{hh}^{\left(f\right)}h_{t-1}+b^{\left(f\right)}\right),$$

(3) $${\tilde{c}}_{t}=tanh\left(W_{ih}^{\left(g\right)}x_{t}+W_{hh}^{\left(g\right)}h_{t-1}+b^{\left(g\right)}\right),$$

(4) $$o_{t}=\sigma \left(W_{ih}^{\left(o\right)}x_{t}+W_{hh}^{\left(o\right)}h_{t-1}+b^{\left(0\right)}\right),$$

(5) $$c_{t}=f_{t}⊙c_{t-1}+i_{t}⊙{\tilde{c}}_{t},$$

(6) $$h_{t}=o_{t}⊙tanh\left(c_{t}\right),$$

where $x_{t}$ represents the input vector at time step T, and $i_{t},f_{t}$, and $o_{t}$ represent the input, forget, and output gates, respectively. The input gate $i_{t}$ regulates how much new information is added to the memory cell, while the forget gate $f_{t}$ determines how much past information should be retained. The output gate $o_{t}$ controls how much of the cell state contributes to the hidden state. The candidate cell state ${\tilde{c}}_{t}$, computed using the hyperbolic tangent function tanh(⋅), represents a potential update to the cell state $c_{t}$, which maintains the memory of the recurrent network. The Hadamard product ⨀ denotes element-wise multiplication, allowing gate values to selectively update specific elements of the memory and hidden states.

The weight matrices $W_{ih}^{\left(i\right)}$, $W_{hh}^{\left(i\right)}$, $W_{ih}^{\left(f\right)}$, $W_{hh}^{\left(f\right)}$, $W_{ih}^{\left(g\right)}$, $W_{hh}^{\left(g\right)}$, $W_{ih}^{\left(o\right)}$, $W_{hh}^{\left(o\right)}$ are learnable parameters that determine how the input and previous hidden state influence the input, forget, candidate, and output gates, respectively. The bias vectors $b^{\left(i\right)},b^{\left(f\right)},b^{\left(g\right)},b^{\left(o\right)}$ introduce additional trainable parameters to each gate, enabling better adaptation to data. The hidden state $h_{t}$ is computed using the updated cell state and the output gate activation. The final hidden state from the last time step in the last LSTM layer serves as a compact representation of the patient’s 24-hour clinical trajectory and is passed through a fully connected layer to produce a single logit. The logit is the raw output before applying the sigmoid function σ(⋅), which maps the logit to a probability score in the range of (0,1). To improve generalization and reduce overfitting, batch normalization and dropout layers are applied after the LSTM layers during training.

Although the raw outputs of neural networks often yield probabilistic predictions, these probabilities may not be well calibrated. To improve the reliability of the predicted probabilities, we applied Platt scaling, a form of calibration that uses a logistic regression model to map the network’s predicted scores to better-aligned probabilities. Specifically, we trained a logistic regression model on the validation set using the network’s output probabilities as input features and the true labels as targets. This calibration step, applied after the LSTM inference is complete, ensures that the final output probabilities more accurately reflect the true likelihood of IWS occurrence. The combination of LSTM based temporal modeling, normalization, regularization, and post hoc calibration thus yields a robust and clinically interpretable risk assessment tool.

### Model Explainability

To address the black box nature of deep learning models, LRP was integrated into the FIR-LSTM architecture. LRP redistributes the final prediction’s relevance back through the network’s layers and time steps, attributing importance scores to each input feature at each epoch. This process transforms the network’s prediction into a clear, quantitative explanation of which variables and time periods were most influential in determining the final IWS risk score. Incorporating LRP into LSTM networks necessitates carefully tracing the model’s predictions back through its recurrent structure. Unlike feed-forward networks, LSTMs process sequential inputs and maintain internal states over multiple time steps. To ensure that each input feature at each time step is assigned an appropriate share of the final prediction’s importance, LRP must redistribute the relevance not only across network layers but also backward through time. In FIR-LSTM, LRP begins assigning relevance to the model output by setting it equal to the model’s final prediction before calibration. This relevance is first backpropagated through a fully connected output layer using standard LRP rules for linear transformations as shown in

(7) $$R^{\left(h_{t}\right)}=\sum_{k}\frac{h_{t}\;\cdot \;\omega_{k}\;+\;\epsilon \;\cdot \;sign\left(h_{t}\;\cdot \;\omega_{k}\right)}{y\;+\;\epsilon }\cdot R^{\left(y\right)},$$

where $R^{\left(h_{t}\right)}$ is the relevance of the last hidden state, $h_{t}$ is the last hidden state, $\omega_{k}$ is the weight between the output layer and the fully connected layer, y represents the logit (i.e., output from the model before calibration), $R^{\left(y\right)}$ is the initial relevance score at the output layer, and ϵ is a stabilizer for numerical stability. The next step decomposes $R^{\left(h_{t}\right)}$ into relevance assigned to the cell state and output gate at time t. Based on Equation (6), we distribute the relevance from $h_{t}$ to $c_{t}$ proportionally to how $c_{t}$ and $o_{t}$ influence $h_{t}$ through

(8) $$\frac{\partial h_{t}}{\partial c_{t}}=o_{t}⊙(1-{\left(tanh\;c_{t}\right)}^{2}),$$

(9) $$R^{\left(c_{t}\right)}=R^{\left(h_{t}\right)}⊙o_{t}⊙(1-{\left(tanh\;c_{t}\right)}^{2}).$$

Having determined $R^{\left(c_{t}\right)}$, the relevance is split between the contributions of the input and forget branches of the cell update. Based on Equation (5), $R^{\left(c_{t}\right)}$ is partitioned according to the relative magnitudes of each branch through

(10) $$R^{\left(f,⊙c_{t-1}\right)}=\frac{f_{t}⊙c_{t-1}}{f_{t}⊙c_{t-1}\;+\;i_{t}⊙{\tilde{c}}_{t}\;+\;\epsilon }\cdot R^{\left(c_{t}\right)},$$

(11) $$R^{\left(i_{t}⊙{\tilde{c}}_{t}\right)}=\frac{i_{t}⊙{\tilde{c}}_{t}}{f_{t}⊙c_{t-1}\;+\;i_{t}⊙{\tilde{c}}_{t}\;+\;\epsilon }\cdot R^{\left(c_{t}\right)}.$$

From here, the relevance is distributed to the candidate gate proportionally as shown in

(12) $$R^{c_{t}}=R^{\left(i_{t}⊙c_{t}\right)}⊙i_{t}.$$

Focusing on the candidate gate when attributing relevance in an LSTM provides a strategically simplified yet meaningful perspective on feature importance analysis. The candidate gate serves as the primary pathway through which new information is integrated into the cell state, while the input, forget, and output gates primarily regulate the retention, modulation, and visibility of existing information. By isolating the candidate gate for LRP analysis, we directly capture the contribution of each input feature at the moment new content is incorporated into the model’s temporal representation. This approach not only preserves the fundamental principle of relevance conservation but also reduces the complexity of disentangling gating mechanisms that predominantly act as regulators rather than direct information carriers. To distribute the relevance proportionally, we apply the following relevance redistribution rule:

(13) $$R_{j}^{\left(h_{t-1},x_{t}\right)}=\frac{a_{j}\;\cdot \;\omega_{j}\;+\;\epsilon \;\cdot \;sign\left(a_{j}\;\cdot \;\omega_{j}\right)}{\sum_{k}\left(a_{k}\;\cdot \;\omega_{k}\;+\;\epsilon \;\cdot \;sign\left(a_{k}\;\cdot \;\omega_{k}\right)\right)}\;\cdot \;R_{j}^{\left(\varepsilon_{t}\right)},$$

where $a_{j}$ represents the input to the candidate gate, formed by the concatenation of the previous hidden state $h_{t-1}$ and the current input vector $x_{t}$. The term $\omega_{j}$ denotes the corresponding weight associated with the candidate gate, while $R_{j}^{\left({\tilde{c}}_{t}\right)}$ represents the relevance assigned to the 𝑗-th component of the candidate cell state. The summation index k in the denominator iterates over all components of, $h_{t-1},x_{t}$ ensuring that the total relevance is normalized across all input dimensions. Thus, the relevance is redistributed to $x_{t}$, yielding an interpretable measure of feature importance.

This procedure repeats recursively, moving from t = 𝑡 to t = 1, at each step redistributing relevance to progressively earlier time points. By the end, each input feature at every time step is assigned a relevance score that reflects its cumulative contribution (i.e., feature importance) to the final prediction. LRP for LSTM layers in FIR-LSTM provides a principled, mathematically grounded method for deconstructing the model’s decision-making process. The approach ensures relevance conservation at every step, rigorously adheres to the forward computational graph, and produces human-interpretable explanations of which features, and which time periods most significantly influenced the model’s predicted risk. This is especially valuable in clinical settings, where understanding the rationale behind a model’s prediction is critical for building trust and guiding more informed clinical decisions.

## Results

### Datasets

The study population used for model development included all pediatric and cardiac intensive care units (ICU) patients at Children’s National Hospital from July 1, 2012, to July 1, 2023, who were less than 22 years of age and were mechanically ventilated and subsequently extubated, encompassing a broad range of diagnostic categories. Other inclusion criteria included intubation and mechanical ventilation, at least one recorded WAT-1 score in the EHR after extubation and removal of mechanical ventilatory support. Neonatal ICU (NICU) patients and those with a tracheostomy were excluded. After data preprocessing, a total of 2,100 patients were included in the experiments, with 1,680 allocated for training and 420 for testing. Moreover, to further evaluate the generalizability of our model for IWS prediction, an independent cohort of patients less than 22 years of age from July 2, 2023, to January 31, 2025, was extracted to perform a prospective validation of FIR-LSTM. This independent dataset comprises 400 new patients and was preprocessed using the same pipeline as the original training data. Table 1 shows the characteristics of all patients used in this study.

The data contained detailed, time-stamped information on vital signs (e.g., heart rate, blood pressure, respiratory rate, temperature), medication administrations (including analgesics and sedatives), ventilatory parameters, and standard demographic variables such as age and prior clinical history of withdrawal, and the dataset was prepared in accordance with institutional guidelines and institutional review board approvals. The resulting cohort offered a comprehensive, high-frequency longitudinal record of patient trajectories in the PICU, forming the basis for subsequent feature extraction, preprocessing, and model development.

### IWS Prediction

FIR-LSTM demonstrated the robust performance in assessing the risk of IWS based on 24-hour longitudinal EHR data. As shown in Table 2, by fixing specific sensitivity values, we explored the trade-offs between sensitivity and precision to identify clinically meaningful performance points. For instance, at a sensitivity of 0.70, the model achieved a precision of 0.45 and an accuracy of 0.79, while maintaining a specificity of 0.81 and a negative predictive value (NPV) of 0.92. As the sensitivity increased, precision and accuracy slightly decreased, reflecting the model’s emphasis on capturing a higher proportion of true positives at the expense of a greater number of false positives. Despite this trade-off, the consistently high NPV across thresholds indicated the model’s strong reliability in ruling out IWS risk when predictions were negative.

**Figure 3.**
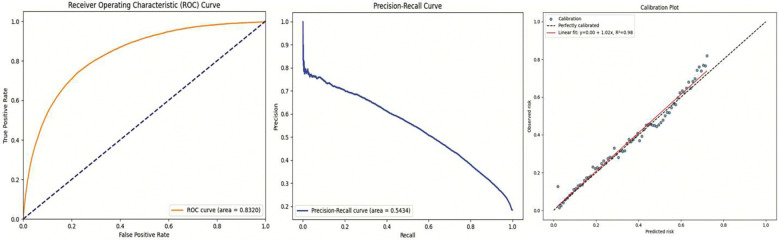
ROC, PR, Calibration curves for the trained FIR-LSTM on IWS prediction.

Beyond threshold-specific metrics, as we can see from Figure 3, the model exhibited a strong Area Under the Receiver Operating Characteristic Curve (AUROC) of 0.83, underscoring its ability to discriminate between children who would develop IWS and those who would not. The Precision-Recall Curve further highlighted the model’s capacity to identify positive cases under class imbalance conditions. Additionally, calibration plots demonstrated that the predicted probabilities were well-aligned with observed outcomes, ensuring that the output scores could be interpreted meaningfully as real IWS risk estimates rather than merely relative rankings.

### Feature Importance

**Figure 4.**
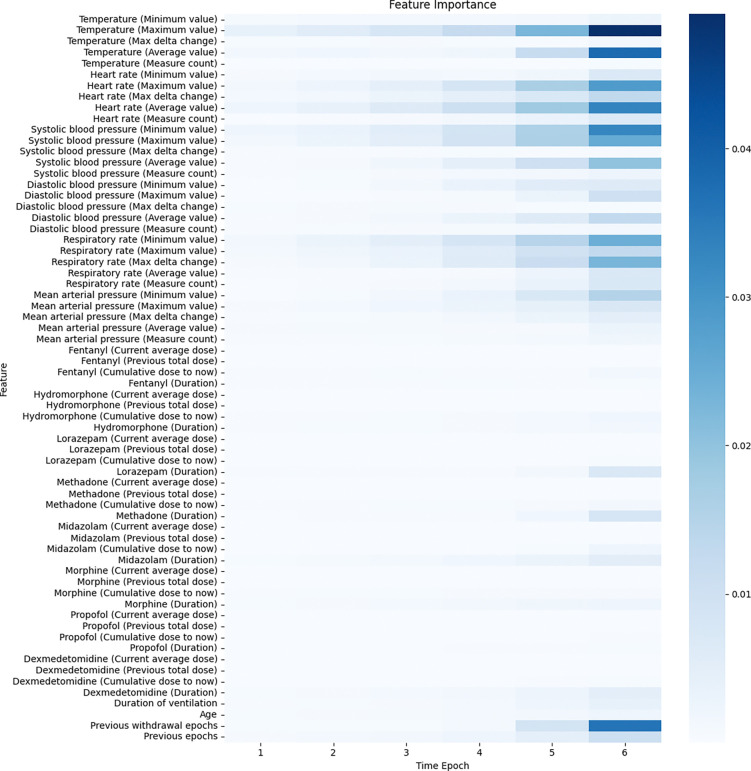
Feature importance on all features and time epochs.

**Figure 5.**
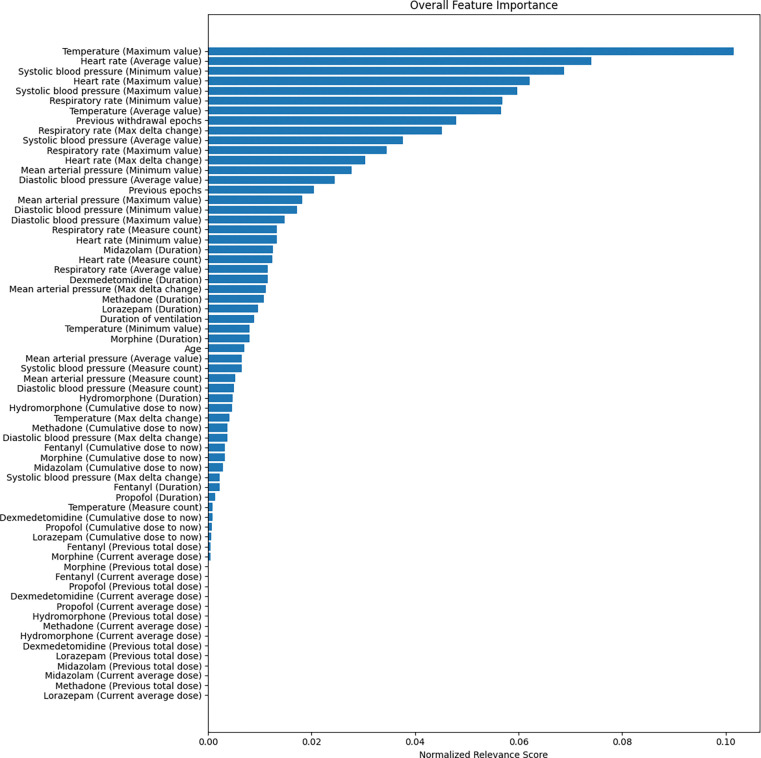
Aggregated feature importance across time steps.

To further elucidate the model’s decision-making process, we employed LRP to derive feature importance scores. Figure 4 shows the importance of each feature at each time epoch from a global view, and Figure 5 shows the aggregated relevance across time steps which enabled a clear, interpretable ranking of feature importance.

From Figure 4 and 5, we can see that vital signs emerged as dominant contributors to IWS risk estimation, with temperature and heart rate measures consistently receiving high relevance scores. Notably, maximum and average values of temperature, as well as the maximum, minimum, and average values of heart rate and blood pressure, were especially influential. This finding aligns with clinical expectations that variability and extremes in vital sign parameters may signal stress or withdrawal-related disturbances. Medication usage patterns also played a role in IWS prediction. Cumulative doses of analgesics and sedatives, as well as the presence of previous withdrawal epochs, showed moderate relevance. The observed temporal patterns indicated that recent measurements had a greater effect on the final prediction, supporting the notion that closer-to-real-time data segments are more predictive of imminent IWS onset.

In summary, the LRP-based interpretability analysis revealed that the model’s predictions are strongly guided by critical clinical parameters such as vital sign fluctuations and recent medication histories. This transparency not only validates the model’s clinical plausibility but also helps clinicians understand which factors may be early harbingers of withdrawal risk, ultimately supporting more targeted and proactive patient management strategies.

### Time Epoch Length Selection

**Figure 6.**
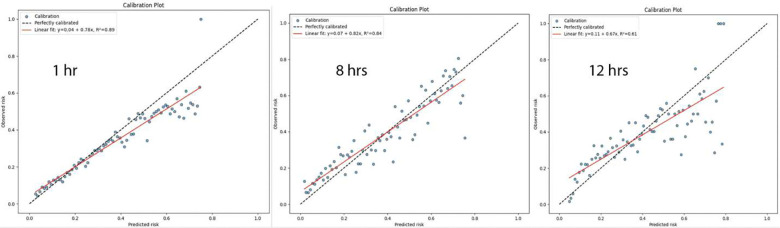
Calibration plots for 1-hour, 8-hour, 12-hour time epoch length.

To assess the impact of the time epoch length on predictive performance, we conducted an evaluation across multiple interval configurations. Specifically, we compared models trained to predict IWS using 1-hour, 4-hour, 8-hour, and 12-hour length of time epochs. Each model still leveraged a 24-hour observation window, composed of a corresponding number of these intervals, to forecast IWS risk in the subsequent time frame. The results shown in Table 3 indicated that a 4-hour epoch length yielded the most balanced and robust performance across key metrics, including AUROC, AUPRC, sensitivity, and precision. Moreover, from Figure 6 we can see that either shorter or longer intervals are associated with a model that is unable to be calibrated, thus unable to reflect the true risk of IWS. Shorter intervals (e.g., one hour) provided finer granularity but led to noisier signals, potentially diluting the ability to detect meaningful variability patterns. Conversely, longer intervals (e.g., eight or 12 hours) diminished the temporal resolution, making it more challenging to capture rapid physiological changes pertinent to emerging withdrawal symptoms. The 4-hour epoch configuration appeared to capture sufficient variability in vital signs without overwhelming the model with excessive temporal fragmentation, thus offering an optimal trade-off between granularity and stability in the input data.

### Look-Back Time Selection

Beyond the length of each time epoch, we examined how far back into the patient’s clinical history the model should look to achieve optimal predictive performance. We chose the 4-hour time epoch length, trained and evaluated models using various observation windows ranging from four to 48 hours, maintaining a consistent epoch length while adjusting the number of epochs accordingly.

Table 4 suggested that both 6-hour epoch (24 hours) and 12-hour epoch (48 hours) observation windows supported strong predictive capabilities, however, applying a look-back time window greater than 8 hours all gave comparatively good performance. Although both the 24-hour and 48-hour observation windows yielded comparable discriminative performance (AUROC = 0.83; AUPRC = 0.54), extending the window to 48 hours posed practical limitations. Specifically, it would delay model applicability until at least 48 hours of patient’s data were available, thereby postponing early risk stratification. Moreover, longer histories tended to overemphasize past withdrawal episodes at the expense of more recent, clinically actionable signals. In contrast, the 24-hour window allowed for earlier deployment without sacrificing performance, and better aligned with clinical reasoning that prioritizes proximal indicators of a patient’s evolving state. Accordingly, we adopted the 24-hour window to maximize both clinical relevance and model usability.

### Model Benchmarking

To further validate the effectiveness of our modeling approach, we benchmarked our FIR-LSTM model against multiple alternative architectures, each representing a distinct strategy for handling sequential and high-dimensional EHR data. Specifically, we trained a temporal convolutional network (TCN), a convolutional neural network (CNN) optimized for time series data (i.e., 1D convolutions), a multilayer perceptron (MLP) that treats sequential inputs as flattened features, a Transformer-based model that leverages attention mechanisms, a gated recurrent unit (GRU) model as a more parameter-efficient RNN variant, basic LSTM architecture, and our proposed FIR-LSTM with 500 epochs by using the same training, validation, and testing data, and the same dropout rate, hidden dimension, and number of layers, and then compared performance of all benchmarking models. Table 5 shows the general statistics of these models when achieving a 0.80 sensitivity.

From Table 5, we can see that the performance underscored the advantages of recurrent-based architectures, with LSTM and FIR-LSTM configurations outperforming other approaches in key evaluation metrics. Both LSTM and FIR-LSTM models demonstrated higher AUROC scores, indicating stronger discriminative power in distinguishing between patients who would develop IWS and those who would not. Similarly, these RNN-based models achieved superior precision, accuracy, and specificity compared to the CNN, MLP, and Transformer models. Among the tested architectures, FIR-LSTM showcased a slight edge over the standard LSTM, achieving a balanced trade-off between sensitivity and precision and thus offering a nuanced understanding of the patient’s condition without inflating the false positive rate. The main reason for the trivial difference is primarily due to how it initializes parameters and handles relevance propagation for interpretability. By carefully defining a custom parameter initialization (i.e., uniform distribution within a certain range based on the hidden dimension), FIR-LSTM can achieve more stable and potentially more expressive internal representations. This improved stability might arise because a more controlled initialization prevents the weights from starting in poorly scaled regions of the parameter space, thereby facilitating smoother training dynamics. Additionally, the FIR-LSTM architecture implements a manually unrolled LSTM to facilitate LRP, whereas PyTorch’s built-in basic LSTM relies on highly optimized kernels for forward and backward passes. As a result, although the overhead from storing gate activations and hidden states at each time step can prolong training time compared to basic LSTM, it enables deeper insights into the model’s decision-making process.

While the TCN, CNN, and MLP models provided competitive training efficiencies and shorter runtimes, their predictive performance fell behind the RNN-based methods. The Transformer model, although adept at capturing long range dependencies, did not substantially surpass simpler architectures, likely due to the data’s relatively short effective sequence length and the complexity of tuning attention-based models for this specific clinical prediction task.

**Figure 7.**
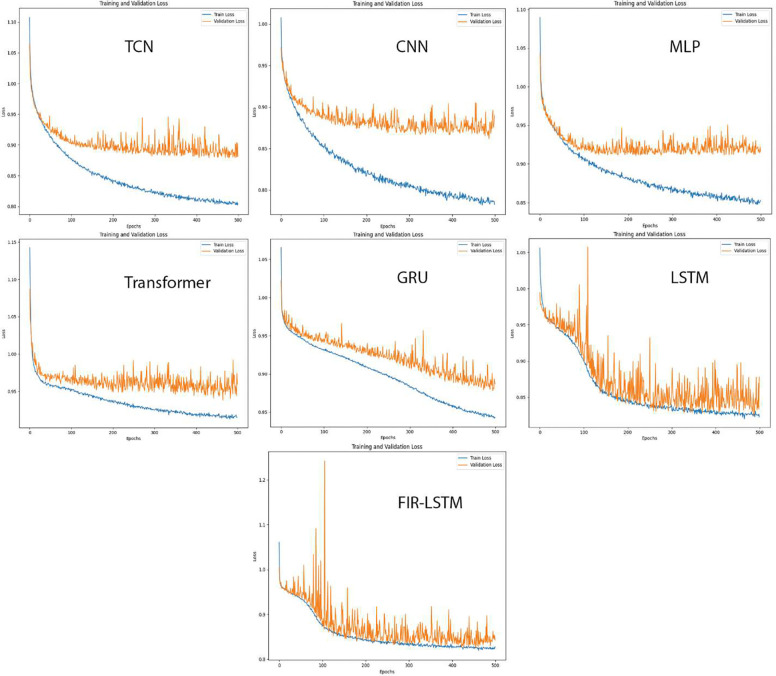
Training and validation loss of all benchmarking models.

Figure 7 illustrates the training loss and the validation loss during the training process of all benchmarking models, which provides insights into the stability and learning dynamics of each architecture. Across models, we observed that RNN-based approaches (LSTM and FIR-LSTM) tended to yield smoother and more consistent convergence patterns, with validation losses stabilizing over extended training epochs without substantial overfitting. This stability suggested that recurrent architectures are well-suited for leveraging temporal structure in EHR data in IWS prediction.

In contrast, models like the CNN, TCN, and MLP often exhibited more pronounced fluctuations in validation loss and had an apparent trend of overfitting, particularly in later epochs. This volatility might reflect their sensitivity to subtle shifts in the data distribution or their reliance on local receptive fields to capture temporal patterns, indicating potential difficulty in capturing sequential nuances without explicit temporal modeling. The Transformer model, although capable of modeling complex dependencies, also showed evidence of overfitting.

Despite the apparent plateau of training and validation loss across all evaluated models at approximately 0.8 to 0.9, this outcome should not be interpreted as a failure to learn clinically meaningful patterns. Rather, it reflects the inherent complexity of predicting IWS, a multifactorial and partially latent condition influenced by diverse physiological, pharmacological, and temporal dynamics. Prior clinical studies have noted that even expert-derived scoring systems like the Withdrawal Assessment Tool - 1 (WAT-1) exhibit substantial inter-observer variability and limited predictive granularity, underscoring the inherent ambiguity in IWS diagnosis^6^.

Importantly, our proposed FIR-LSTM model demonstrates superior convergence stability, with a consistently lower and smoother validation loss trajectory compared to benchmarking architectures such as standard LSTM, GRU, Transformer, and CNN models. This suggests better generalization and robustness to temporal noise and irregularity in multivariate EHR sequences. While the absolute loss appears to saturate across models, this saturation point likely reflects the maximum attainable signal fidelity given the current data resolution, not a lack of model capability.

The model was well-calibrated, as evidenced by the calibration plot in Figure 3. This implies that the output probabilities reflect true underlying risk, an important requirement for clinical applicability. Our calibrated output supports risk stratification and threshold-based intervention in a real-world clinical setting. Finally, our interpretability framework (via LRP) reveals that the model captures physiologically plausible and temporally coherent signals, offering mechanistic insights beyond simple classification and risk prediction. Together, these findings suggest that despite the absence of near-zero loss—which is biologically implausible in this context—FIR-LSTM adds value by improving clinical decision support through calibrated risk estimates, generalizing better than alternative architectures, and providing interpretable evidence of feature-timepoint interactions associated with IWS onset.

### Feature Importance Validation

While our initial LRP analysis identified a range of features that strongly influenced the model’s predictions, a key step in solidifying confidence in these results involves formal validation of feature importance. We employed permutation-based tests to quantify how perturbing individual features affects the model’s performance. Importantly, to avoid confounding factors and ensure the clarity of interpretation, we focused specifically on a subset of important monotonically varying features such as Previous withdrawal epochs, Previous epochs and Midazolam (Duration). These features were selected despite not necessarily being the most dominant drivers of the prediction. Instead, their monotonic behavior allowed us to more directly evaluate the effect of changes in their values on the model’s prediction.

In this validation analysis, two forms of permutation-based assessment were conducted to quantify feature importance. First, we conducted global permutations, in which the values of each selected feature were randomly shuffled across the entire 24-hour observation window. This strategy enabled us to evaluate the cumulative impact of individual features on overall model performance. As summarized in Table 6, we focused on permuting monotonic features and assessed their influence on predictive accuracy using the model’s raw, uncalibrated outputs. Predictions were binarized using the default threshold of 0.5. Performance degradation following permutation consistently aligned with the monotonic contribution of these features—either positively or negatively—to the model’s predictions. This direct approach offers insight into the model’s internal attribution of relevance, independent of post-hoc calibration. Notably, permuting the Previous withdrawal epochs led to a pronounced decline in performance, highlighting the model’s reliance on the temporal accumulation of withdrawal episodes to assess impending risk. Comparatively, permuting Previous epochs or Midazolam (Duration) resulted in modest declines in performance, suggesting that stable temporal patterns in medication exposure or the progression of time epochs themselves also carry predictive significance, though to a lesser extent than prior withdrawal episodes.

Second, we performed time-specific permutations, targeting features at specific time steps. This granular approach uncovered that the predictive contribution of these features can vary considerably across time epochs. From Figure 4, we can see that Previous withdrawal epochs at time epoch 6 is the more important than time epoch 5, and Previous epochs is comparatively less important. Hence, by selectively shuffling values at a particular epoch (e.g., the fifth- or sixth-time epoch), we examined whether the model places disproportionate weight on recent versus distant historical signals. Table 7 shows the performance of this permutation validation. The resultant performance drop for features like Previous withdrawal epochs at later epochs emphasized that the model indeed assigns higher relevance to temporally closer data points. In other words, while a monotonic feature may hold clinical relevance throughout the sequence, its immediate past values appear more critical for forming an accurate near-future risk assessment.

In choosing monotonic features for these validations, we intentionally concentrated on variables whose directionality and magnitude shifts are relatively straightforward to interpret. Although these features were not always the top-ranked variables from the LRP analysis, their monotonic nature simplifies the attribution process: any distortion introduced by permutation is more directly interpretable as a break in a discernible trend rather than a complex interplay of irregular fluctuations. Consequently, the observed decreases in performance upon permuting these features serve as a robust, conceptually clean confirmation that the model’s reliance on them is nonspurious and aligned with the model’s logic inferred via LRP.

In summary, by carefully selecting monotonic features for permutation-based validation, we can demonstrate that the feature importance results align with a coherent interpretation of how the model processes temporal data. The resulting performance degradations upon permutation provide further evidence that the identified important features are indeed meaningful contributors to the prediction, reinforcing the reliability of our LRP-derived feature importance explanations.

### Independent Cohort Validation

**Figure 8.**
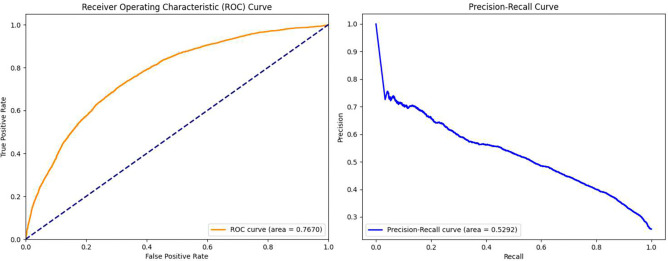
ROC, PR curves for the FIR-LSTM on the independent validation dataset.

To further evaluate the generalizability of our model for IWS prediction, an independent cohort of patients less than 22 years of age from July 2, 2023, to January 31, 2025, was extracted to perform a prospective validation of FIR-LSTM. This independent dataset comprises 400 new patients and was preprocessed using the same pipeline as the original training data, yielding a total of 17,739 datapoints for validation. Figure 8 presents the ROC and PR curves for the model’s performance on the dataset. Notably, the model demonstrates stable performance compared to the original dataset, with an improvement in the AUPRC. This enhancement is likely attributable to a higher IWS prevalence in the new dataset—0.34 versus 0.23 in the original cohort.

In addition, Table 8 summarizes the performance metrics of FIR-LSTM across varying sensitivity thresholds on the independent validation dataset, from where we can see that despite the shift in label distribution, the model demonstrates robust and consistent performance across a range of sensitivity thresholds. As sensitivity increases from 0.70 to 0.95, precision and specificity gradually decrease, as expected. However, the F1-score remains stable—ranging from 0.54 at sensitivity 0.70 to 0.47 at sensitivity 0.95—closely matching the performance observed on the original dataset. Negative predictive value (NPV) also remains high (0.87–0.94), reinforcing the model’s strong reliability in ruling out IWS. These metrics indicate that the model effectively balances sensitivity and precision, even under conditions with increased positive case prevalence. Importantly, the model achieves a slightly higher AUPRC on the new dataset compared to the original, likely due to the increased proportion of IWS cases. This improvement, coupled with the stable F1-scores and high NPV, supports the model’s generalizability with varying prevalence profiles.

## Discussion

In this study, we introduced FIR-LSTM, a novel interpretable deep learning framework for predicting IWS in critically ill pediatric patients using longitudinal EHRs. This work addresses a critical gap in current clinical practice, where IWS is typically assessed using the WAT-1 scoring tool—a method that, despite its widespread use in North America, is inherently subjective and reliant on manual evaluations. Such limitations can delay timely risk identification and appropriate clinical response. By contrast, FIR-LSTM offers an automated, data-driven approach that enables early and accurate prediction of IWS risk, supporting proactive clinical decision-making. To our knowledge, this is the first study to propose a deep learning model specifically designed to aid in IWS risk assessment in pediatric ICU patients, thereby laying the groundwork for more efficient and effective withdrawal management protocols.

Through comprehensive benchmarking against a suite of state-of-the-art models—including convolutional, feedforward, recurrent, and attention-based architectures—we show that recurrent neural networks, and particularly LSTM-based models, remain highly effective for this temporal prediction task. FIR-LSTM not only achieved consistently lower training and validation losses but also exhibited superior convergence stability compared to Transformer- and CNN-based baselines. Importantly, we systematically optimized the temporal resolution and observation window, finding that a 4-hour epoch with a 24-hour lookback horizon most effectively captures predictive clinical patterns.

A key contribution of FIR-LSTM is its integration with LRP, which facilitates granular interpretability across both time and feature dimensions. This mechanistic transparency not only aligns model predictions with clinical intuition—highlighting the influence of cumulative withdrawal history, medication history and key physiological markers—but also empowers domain experts to interrogate, validate, and refine model behavior. On the other hand, although traditional performance evaluation metrics such as F1-score remain modest—a reflection of both label noise and the clinical complexity of IWS—this should not obscure the practical value of the model. FIR-LSTM is well calibrated, ensuring that predicted probabilities closely track empirical outcome rates. In the clinical setting, this calibration enables reliable risk stratification, supporting decisions such as anticipatory monitoring, medication tapering, or early intervention^27,28^, all of which require nuanced risk awareness rather than binary prediction alone. In this regard, the model’s outputs serve as a probabilistic decision support tool, enabling flexible thresholding based on clinician discretion or institutional protocol.

Together, our results support the conclusion that predictive modeling of IWS is feasible and clinically valuable, even in the presence of temporal uncertainty and outcome heterogeneity. FIR-LSTM offers a scalable, interpretable, and calibrated approach to anticipating withdrawal risk, with direct implications for personalized sedation weaning strategies, resource allocation, and early identification of vulnerable patients. Future work may extend this framework to incorporate additional context such as clinical notes or caregiver observations, enabling even richer representations of withdrawal risk and patient state. In addition, incorporation of the model into bedside practice may allow further improvement of the model based on the clinical feedback.

## Supplementary Material

Supplementary Files

This is a list of supplementary les associated with this preprint. Click to download.

AppendixA.docx

AppendixB.docx

## Figures and Tables

**Table 1.** Characteristics of pediatric ICU patients who had an episode of IWS following mechanical ventilation.

Characteristics	Development dataset (1) (n = 2100)	Prospective validation dataset (n = 400)
Age in Months (median hours (IQR))	15.0 (4.0 – 67.25)	18.3 (3.3 – 87.1)
≤1 months	256 (12.2%)	58 (14.5%)
> 1mo – < 2 years	978 (46.6%)	158 (39.5%)
≥ 2 – <6 years	359 (17.1%)	70 (17.5%)
≥6 – <13 years	269 (12.8%)	65 (16.3%)
≥13 – <22years	238 (11.3%)	49 (12.3%)
Female (N (%))	961 (45.8%)	211 (52.8%)
Race (N (%))		
Caucasian	431 (20.5%)	77 (19.3%)
African American	777 (37.0%)	139 (34.8%)
Unknown/Other	892 (42.5%)	184 (46.0%)
Hospital Length of Stay (median hours (IQR))	526.1 (311.5 – 957.4)	522.3 (301.2 – 953.7)
ICU Length of Stay (median hours (IQR))	422.7 (201.2 – 1026.9)	410.3 (196.1 – 1011.3)
Duration of Mechanical Ventilation (median hours (IQR))	194.2 (111.1 – 383.4)	196.2 (102.6–500.7)
Hospital Mortality (N (%))	264 (12.6%)	42 (10.5%)
Time From Extubation to First Episode of IWS (median hours (IQR))	5.0 (1.0 – 28.0)	7.0 (1.0 – 36.0)
Opioid Medications (N (%))		
Fentanyl	1921 (91.5%)	386 (96.5%)
Morphine	1647 (78.4%)	308 (77.0%)
Hydromorphone	539 (25.7%)	88 (22.0%)
Methadone	1225 (58.3%)	229 (57.3%)
Sedative Medications (N (%))		
Midazolam	2014 (95.9%)	347 (86.8%)
Dexmedetomidine	1523 (72.5%)	387 (96.8%)
Lorazepam	1364 (65.0%)	205 (51.3%)
Propofol	1135 (54.1%)	242 (60.5%)

(1). Development dataset consisted of training and testing datasets.

(2). Abbreviations: IWS = Iatrogenic Withdrawal Syndrome. IQR = interquartile range.

**Table 2.** Statistics of FIR-LSTM with different sensitivity values on IWS prediction.

Sensitivity	Precision	Accuracy	Specificity	Negative Predictive Value (NPV)	F1-score
0.70	0.45	0.79	0.81	0.92	0.55
0.75	0.42	0.76	0.77	0.93	0.54
0.80	0.38	0.73	0.71	0.94	0.52
0.85	0.34	0.68	0.64	0.95	0.49
0.90	0.31	0.61	0.54	0.96	0.46
0.95	0.26	0.51	0.41	0.97	0.41

**Table 3.** AUROC and AUPRC of FIR-LSTM with different time epoch length

Time Epoch Length (hour)	AUROC	AUPRC
1	0.77	0.43
4	**0.83**	**0.54**
8	0.76	0.50
12	0.73	0.43

**Table 4.** AUROC and AUPRC of FIR-LSTM with different look-back time for future prediction

Look-Back Time (hours)	AUROC	AUPRC
4	0.74	0.39
8	0.76	0.42
12	0.81	0.52
16	0.82	0.54
20	0.82	0.52
24	**0.83**	**0.54**
28	0.80	0.53
32	0.82	0.52
36	0.82	0.50
40	0.81	0.52
44	0.81	0.49
48	**0.83**	**0.54**

**Table 5.** Statistics of performance on different benchmarking models achieving a 0.80 sensitivity

Model	AUROC	AUPRC	Precision	Accuracy	Specificity	F1-score	NPV	Run Time (hours)
TCN	0.73	0.35	0.29	0.59	0.55	0.42	0.92	1.05
CNN	0.73	0.36	0.29	0.59	0.55	0.42	0.92	0.62
MLP	0.74	0.37	0.30	0.61	0.57	0.43	0.93	**0.43**
Transformer	0.75	0.37	0.30	0.62	0.58	0.44	0.93	1.67
GRU	0.77	0.41	0.31	0.63	0.59	0.44	0.93	0.70
LSTM	0.81	0.49	0.35	0.69	0.67	0.49	**0.94**	0.76
FIR-LSTM	**0.83**	**0.54**	**0.38**	**0.73**	**0.71**	**0.52**	**0.94**	1.42

**Table 6.** Statistics of performance on global permutation of top 3 most important monotonic features

Permuted Feature	AUROC	AUPRC	Sensitivity	Precision	Accuracy	Specificity	NPV	F1-score
N/A	0.83	0.54	0.73	0.43	0.77	0.78	0.93	0.54
Midazolam (Duration)	0.81	0.49	0.71	0.40	0.75	0.76	0.92	0.51
Previous epochs	0.77	0.41	0.70	0.37	0.73	0.74	0.92	0.49
Previous withdrawal epochs	0.57	0.26	0.49	0.25	0.63	0.66	0.85	0.33

**Table 7.** Statistics of performance on permutation-based on specific time epochs of top 2 monotonic features

Permuted Feature	AUROC	AUPRC	Sensitivity	Precision	Accuracy	Specificity	NPV	F1-score
Previous epochs at time epoch 5	0.80	0.48	0.70	0.41	0.76	0.77	0.92	0.52
Previous withdrawal epochs at time epoch 5	0.77	0.43	0.68	0.38	0.73	0.75	0.91	0.48
Previous withdrawal epochs at time epoch 6	0.63	0.31	0.57	0.30	0.68	0.70	0.88	0.39

**Table 8.** Statistics of FIR-LSTM presented at different fixed sensitivity values in the independent validation cohort.

Sensitivity	Precision	Accuracy	Specificity	Negative Predictive Value (NPV)	F1-score
0.70	0.45	0.70	0.70	0.87	0.54
0.75	0.42	0.68	0.65	0.88	0.54
0.80	0.40	0.65	0.59	0.89	0.53
0.85	0.38	0.61	0.53	0.91	0.52
0.90	0.35	0.54	0.42	0.92	0.50
0.95	0.31	0.45	0.29	0.94	0.47

## Data Availability

De-identified data is available upon request per hospital policy.

## Code availability

The architecture of FIR-LSTM is freely available in the GitHub repository (https://github.com/haoqiusong/FIR-LSTM) and is released under the MIT license.
